# Deep Learning for the Classification of Non-Hodgkin Lymphoma on Histopathological Images

**DOI:** 10.3390/cancers13102419

**Published:** 2021-05-17

**Authors:** Georg Steinbuss, Mark Kriegsmann, Christiane Zgorzelski, Alexander Brobeil, Benjamin Goeppert, Sascha Dietrich, Gunhild Mechtersheimer, Katharina Kriegsmann

**Affiliations:** 1 Department of Hematology, Oncology and Rheumatology, University of Heidelberg, 69120 Heidelberg, Germany; georg.steinbuss@med.uni-heidelberg.de (G.S.); sascha.dietrich@med.uni-heidelberg.de (S.D.); 2 Institute of Pathology, University of Heidelberg, 69120 Heidelberg, Germany; mark.kriegsmann@med.uni-heidelberg.de (M.K.); christiane.zgorzelski@med.uni-heidelberg.de (C.Z.); alexander.brobeil@med.uni-heidelberg.de (A.B.); benjamin.goeppert@med.uni-heidelberg.de (B.G.); gunhild.mechtersheimer@med.uni-heidelberg.de (G.M.); 3 Translational Lung Research Centre Heidelberg, Member of the German Centre for Lung Research (DZL), 69120 Heidelberg, Germany

**Keywords:** deep learning, artificial intelligence, DLBCL, CLL/SLL, histopathology, CNN

## Abstract

**Simple Summary:**

Histopathological examination of lymph node (LN) specimens allows the detection of hematological diseases. The identification and the classification of lymphoma, a blood cancer with a manifestation in LNs, are difficult and require many years of training, as well as additional expensive investigations. Today, artificial intelligence (AI) can be used to support the pathologist in identifying abnormalities in LN specimens. In this article, we trained and optimized an AI algorithm to automatically detect two common lymphoma subtypes that require different therapies using normal LN parenchyma as a control. The balanced accuracy in an independent test cohort was above 95%, which means that the vast majority of cases were classified correctly and only a few cases were misclassified. We applied specific methods to explain which parts of the image were important for the AI algorithm and to ensure a reliable result. Our study shows that classifications of lymphoma subtypes is possible with high accuracy. We think that routine histopathological applications for AI should be pursued.

**Abstract:**

The diagnosis and the subtyping of non-Hodgkin lymphoma (NHL) are challenging and require expert knowledge, great experience, thorough morphological analysis, and often additional expensive immunohistological and molecular methods. As these requirements are not always available, supplemental methods supporting morphological-based decision making and potentially entity subtyping are required. Deep learning methods have been shown to classify histopathological images with high accuracy, but data on NHL subtyping are limited. After annotation of histopathological whole-slide images and image patch extraction, we trained and optimized an EfficientNet convolutional neuronal network algorithm on 84,139 image patches from 629 patients and evaluated its potential to classify tumor-free reference lymph nodes, nodal small lymphocytic lymphoma/chronic lymphocytic leukemia, and nodal diffuse large B-cell lymphoma. The optimized algorithm achieved an accuracy of 95.56% on an independent test set including 16,960 image patches from 125 patients after the application of quality controls. Automatic classification of NHL is possible with high accuracy using deep learning on histopathological images and routine diagnostic applications should be pursued.

## 1. Introduction

Non-Hodgkin lymphoma (NHL) is a group of hematological neoplasms and among the 10 most common cancer subtypes worldwide [1]. A total of 77,240 new cases and 19,940 cancer-related deaths were estimated to be due to NHL in the United States in 2020, according to the Surveillance, Epidemiology, and End Results (SEER) database [2]. The group of neoplasms constituting NHL is heterogeneous with very different clinical features and variable outcomes of the respective subtypes [3].

The diagnosis and the subtyping of NHL are challenging and require clinical, serological, morphological, and potentially cytogenetic/molecular information. Often, histopathological workup of a lymph node (LN) resection specimen is needed for definite subtyping. As the workup is commonly expensive and time-consuming, a stepwise approach is advocated [4], where thorough morphological evaluation is key and directs the decision on which immunohistochemical and molecular tests need to be performed [5].

Currently, this subjective decision is made by an experienced hematopathologist. However, there are general problems that are expected to aggravate this approach in the future; the overall number of pathologists is decreasing, specifically in Germany, while the overall requirements in terms of knowledge and specialization are increasing [6]. Additionally, not all pathologists can rely on extensive hematopathological experience, as well as expensive and methodological equipment that allows for liberal use of molecular analyses [7,8]. Thus, supplemental methods that support morphological-based decision making and potentially entity subtyping are desirable and needed.

Digital pathology has emerged as an important tool, not only to review histopathological slides, but also to use additional computer-assisted software to support routine diagnostics and research [9]. It has previously been shown that subtyping of carcinoma is feasible [10,11,12,13]. However, few reports are available on the classification of hematological neoplasms, particularly NHL subtypes [14,15,16,17,18]. Therefore, we set out to investigate whether the classification of tumor-free LNs, nodal small lymphocytic lymphoma/chronic lymphocytic leukemia (SLL/CLL), and nodal diffuse large B-cell lymphoma (DLBCL) is possible using deep learning techniques on scanned histopathological slides.

## 2. Materials and Methods

### 2.1. Patient Cohort, Tissue Microarray Construction, and Scanning of Tissue Slides

A cohort of 629 patients was assembled from the archive from the Institute of Pathology, University Clinic Heidelberg, with the support of the Tissue Biobank of the National Center for Tumor Diseases (NCT). The study was approved by the local ethics committee (study number: #S315–2020). While lymph node lymphoma specimens were collected from different anatomical regions, tumor-free lymph node specimens were collected from resection specimens operated for a non-lymphoma tumor disease (lung, colon, and pancreas). Diagnoses of lymphomas were made according to the 2016 World Health Organization Classification of Tumors of Hematopoietic and Lymphoid Tissue [3]. Conventional hematoxylin and eosin staining, as well as immunohistochemistry according to current best practice recommendations, was performed [3]. Tissue microarrays (TMAs) were constructed and subsequently scanned at 400× magnification using a slide scanner (Aperio SC2, Leica Biosystems, Nussloch, Germany) as previously described [19].

### 2.2. Tumor Annotation and Image Patch Extraction

**Figure 1:**
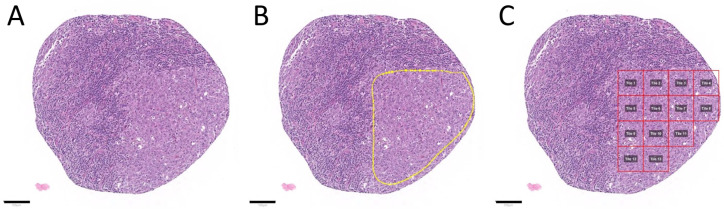
Tumor annotation and generation of image patches. Representative tissue microarray core of a diffuse large B-cell lymphoma without (**A**) and with annotation (**B**)—yellow outline, as well as after image patche creation (**C**)—red squares. The image patches were subsequently saved as .png files. Tile numbers (Tile 1, Tile 2, etc.) are shown in a gray box within each red square in this example. Scale bars (black line): 100 µm.

**Figure 2:**
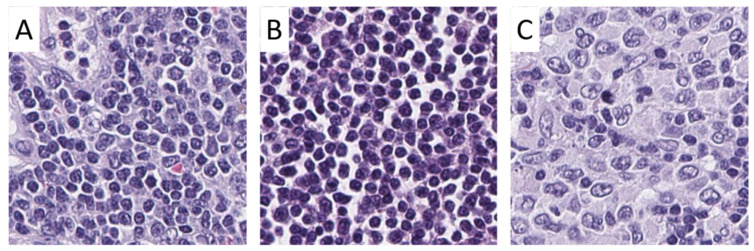
Examples of image patches from annotated areas. Representative image patches from control lymph nodes (**A**), small lymphocytic lymphoma/chronic lymphocytic leukemia (**B**), and diffuse large B-cell lymphoma (**C**) are shown. Magnification: each image 100 × 100 µm (395 × 395 px).

Scanned slides were imported into QuPath (v.0.1.2, University of Edinburgh, Edinburgh, UK). Tumor areas of control LNs, SLL/CLL, and DLBCL were annotated by a pathologist. Patches 100 × 100 µm (395 × 395 px) in size were generated within QuPath [20], and the tumor-associated image patches were exported to the local hard drive. To ensure adequate representation of each tumor, the goal of exporting a minimum of 10 patches per patient was set. Care was taken not to annotate beyond the border of the tissue cores to avoid a prominent representation of the tissue edge, because we anticipated interference with translation of the algorithm to whole slides. Representative tumor areas, tumor annotations, generated patches, and extracted patches are displayed in Figure 1 and Figure 2.

### 2.3. Hardware and Software

For training and prediction with our models, we used the BwForCluster MLS&WISO Production nodes [21] that feature the Nvidia Tesla K80 (models B0 to B3, see also Model training and optimization) or the Nvidia GeForce RTX 2080Ti (model B4). With the Nvidia Tesla K80 nodes, we used both GPUs with a mirrored strategy from TensorFlow. With the Nvidia GeForce RTX 2080Ti nodes, we used a single GPU. Furthermore, we applied singularity (Sylabs, https://sylabs.io/singularity/; v3.7.2, accessed on 1 May 2021) to adopt (v3.6.4) and run (v3.0.1) the TensorFlow 2.3.1-gpu docker container for training and prediction with our models. We added R (v4.0.3) with packages dplyr (v1.0.4), tidyr (v1.1.2), tibble (v3.0.6), config (v0.3.1), readbitmap (v0.1.5), data.tree (v1.0.0), jsonlite (v1.7.2), and jpeg (v0.1–8.1), as well as the python packages pandas (v1.1.5), Pillow (v 8.1.0), scipy (v 1.5.4), tabulate (v0.8.7), and tensorflow_addons (v 0.12.1), to the container. The SmoothGrad heatmaps were generated on a Lenovo P1 Gen 2 running Windows 10 with a conda (v4.9.1) environment containing tensorflow (v2.3.1) and tf-explain (v0.3.0). We used a noise level of 0.5% and a sample size of 50 for SmoothGrad. We adopted the tf-explain package code such that the resulting heatmaps were normalized to [0, 1), and the maximum gradient per each channel was used as described in the respective git repository (https://github.com/sicara/tf-explain/issues/157, accessed on 1 May 2021).

### 2.4. Analytical Subsets

To ensure reliable results, patients were randomly separated into three subsets: training (60%), validation (20%), and test sets (20%). All image patches from a patient (case) were used in the respective subset. We had a checkpoint in our code to ensure that cases were used in a single subset only. These subsets were not changed during the analyses.

### 2.5. Model Training and Optimization

We used models from the EfficientNet family [22] for our analysis. The EfficientNet family is composed of multiple models (from B0 to B7), which are each scaled versions of the baseline model B0. The models were scaled by the compound scaling method introduced in [22]. With compound scaling, each consecutive model increased in network width, depth, and image resolution by a set of fixed scaling coefficients. This form of scaling utilizes the idea that network width, depth, and image resolution seem to exhibit a certain relationship [22]. A model with fewer trainable weights can be trained using fewer resources, and its inference is faster [22]. In this study, we investigated up to which stage the compound scaling did seem to be beneficial in predicting the NHL on histopathological images. The nontrainable model parameters (such as dropout) provided in the tensorflow implementation of EfficientNet models were used without modification. The batch size was chosen as the maximal allowed value (in the sequence of 2^n^, n∈ℕ), given the available GPU memory. The batch size usually becomes smaller when scaling up an EfficientNet; the image resolution increases and the model itself becomes bigger due to the additional weights. We used the Adam optimizer with a learning rate that was selected for each model as follows: models were trained for 50 epochs (each a pass of the full training data) with various learning rates roughly in the range of 10^−5^ to 10^−6^. Then, the best-performing learning rate was chosen, and the respective model was trained further until there seemed to be no further performance gain. Performance was visually evaluated by the achieved validation and training accuracy, the amount of overfitting (difference of training and validation accuracy), and the smoothness of the accuracy curves. The models with highest validation accuracy for each class of EfficientNet models (B0–B4) were compared, and the overall best-performing one was used to classify the test set.

For the tumor-free reference cases, a detailed classification (into LNs from lung, colon, and pancreas) was available, which we used for training of the classifier (we anticipated that this might improve accuracy). Since such a detailed classification was not available for the tumor cases, we analyzed our predictions on the test data using an aggregated class “tumor-free reference LN”.

## 3. Results

### 3.1. Patient Cohort, Annotation, Image Patch Extraction, and Subset Analysis

Cases from SLL/CLL (*n* = 129) and DLBCL (*n* = 119), as well as control LNs from lung, colon, and pancreas (*n* = 381), were identified, retrieved, assembled in a TMA, stained, and scanned. Identification of representative regions resulted in a total of 84,139 extracted 100 × 100 µm (395 × 395 px) image patches. The number of extracted image patches is displayed in Table 1. The goal to extract a minimum of 10 image patches per patient was met in all but seven cases.

### 3.2. Convolutional Neuronal Network Selection and Hyperparameter Optimization

**Figure 3:**
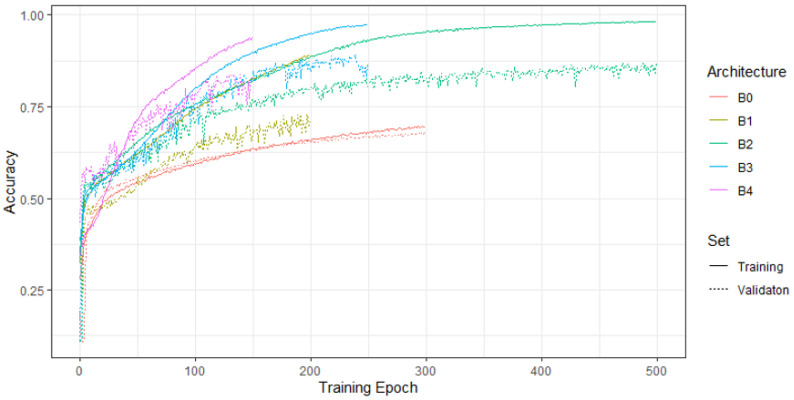
Training and validation accuracy of the model with the highest validation accuracy as per EfficientNet.

Different models (B0–B4) were trained and optimized using different learning rates. Figure 3 shows the training and validation accuracy of the models with the highest validation accuracy per EfficientNet architecture (B0, B1, etc.). Since the B4 architecture did not seem to outperform the B3 architecture, we did not tune the architectures B5–B7 on our data. For the tuned models in Figure 3, the chosen learning rate and batch size were as follows: B0, 1 × 10^−6^, 256; B1, 1 × 10^−5^, 128; B2, 9 × 10^−6^, 128; B3, 8 × 10^−6^, 64; B4, 6 × 10^−6^, 16. Whereas the overall accuracies of the B3 and B2 models were almost on par, the respective confusion matrices on the validation data of the B3 model were slightly more accurate. Thus, we chose the B3 model to classify the test set.

### 3.3. Evaluation of the Test Set and Quality Control

**Figure 4:**
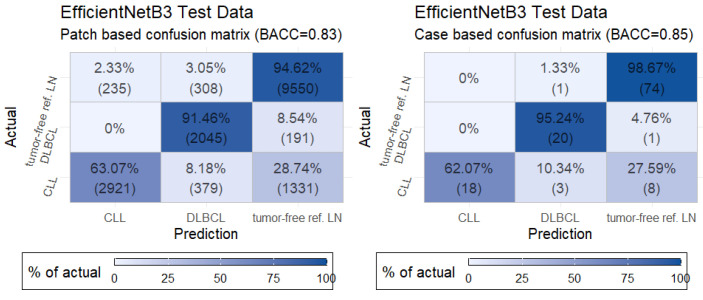
Confusion matrix of the best-performing model in terms of the test data at the patch (**left**) and case level (**right**). The lower panels exhibit the balanced accuracy (BACC). CLL: chronic lymphocytic leukemia, DLBCL: diffuse large B-cell lymphoma, LN: lymph node.

Figure 4 displays the normalized confusion matrix of the selected B3 model in terms of the image patches or cases. For these matrices, image patches were assigned the predicted class with the highest probability, and cases were assigned the predicted class of the majority of their patches. We used the balanced accuracy (BACC) [23] instead of the plain accuracy to account for class imbalance. The model showed a high BACC for DLBCL and the tumor-free reference (in both cases, only a single missed case was identified; Figure 4). However, the predictions for CLL displayed a lower BACC with multiple misclassifications.

Table 2 features the BACC for different quality control thresholds at the case or patch level. Any patch with a predicted probability (in terms of the highest prediction probability) of less than the patch-based quality control (PQC) threshold was filtered out. The case-based quality control (CQC) threshold filtered cases in which the proportion of patches for the predicted class was less than the threshold. From Table 2, one can see that an increase in the case-based quality control threshold improved the overall BACC up to 95.56%. A more detailed example for results with a PQC and CQC of 0.9 showed a decrease in the proportion of misclassified patches and cases (Figure S1). Only 3/102 patients were misclassified using high-quality control thresholds.

**Figure 5:**
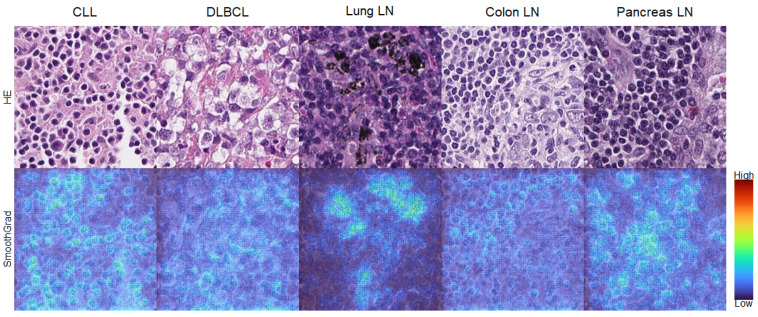
SmoothGrad heatmaps of exemplary patches that were classified correctly. For each class, the upper plot shows the original image patch while the lower plot shows the patch overlaid with the SmoothGrad heatmap with respect to the class of the patch. High SmoothGrad activity scores can be seen in areas overlaid with single cells, as well as in lung LN with extracellular anthracosis. This confirms that the algorithm classified the image patches on the basis of cellular and extracellular morphological structures. DLBCL: diffuse large B-cell-lymphoma, LN: lymph node, SLL/CLL: small lymphocytic lympho-ma/chronic lymphatic leukemia. Magnification: each image 100 × 100 µm (395 × 395 px).

To further investigate if the network was learning the correct features on cells, we applied SmoothGrad [24] to a selection of patches (Figure 5). SmoothGrad produced heatmaps indicating the importance of certain pixels toward the prediction of a certain class. In the heatmaps in Figure 5, we can observe high activity in the respective cells and not in noncellular structures. Thus, we concluded that our model predicted the respective class on the basis of cell morphology.

To estimate the inference latency of our model on a CPU and GPU, we classified a random image patch (each pixel from a uniform distribution) multiple times. We predicted 1000 steps of our final model with a tf.data (https://www.tensorflow.org/guide/data, accessed on 1 May 2021) pipeline using a batch size of 1 that repeated the random image patch. The prediction took 203 s with 203 ms per step (i.e., per image patch) on a single thread of an Intel(R) Core(TM) i9-9880H CPU (2.3 GHz) (Intel Corporation, Santa Clara, USA), and 107 s with 107 ms per step on an Nvidia Quadro T2000 (Nvidia Corporation, Santa Clara, CA, USA).

## 4. Discussion

In the present study, we evaluated and optimized a convolutional neuronal network (CNN) for the classification of histopathological images of tumor-free LNs, SLL/CLL, and DLBCL. The principal capacity of CNN for the classification of malignant and benign diseases on scanned histopathological tissue of conventionally stained sections was previously demonstrated and is well documented [19,25,26,27,28]. Specifically, the technique has been shown to be capable of classifying carcinoma subtypes and of identifying LN metastases of carcinomas [19,29,30,31]. However, studies on the classification of lymphomas are relatively scarce, and normal LNs as controls have rarely been included [14,17,18,22,32,33]. In addition to the classification of lymphoma subtypes, it has been shown that molecular alterations may be detected by deep learning algorithms on histopathological tissue sections [17].

The abovementioned studies on lymphoma classification have in common that they showed that lymphoma subtyping is possible with high accuracy (often >90%) using deep learning techniques when 2–4 lymphoma subtypes are included for classification. In this regard, our study is comparable as we included normal LNs as a control and two common NHL subtypes of B-cell lineage. In line with these previous studies, our BACC was >95%.

Direct comparison of the different studies in terms of methodology is somewhat difficult, as, in addition to the included entities, the number of cases, the study design, the image input parameters, the architecture of the respective networks, and the evaluation were highly heterogeneous.

Commonly, deep learning studies require a large set of images, but there is no consensus on the minimum number of cases that should be included. The previously reported studies on lymphoma subtyping included between 34 and 259 cases per entity and a total of 2560 to 850,000 image patches [14,22,32]. One study included 867 DLBCL cases, but their algorithm was mainly designed to separate DLBCL and samples not related to lymphoma [16]. In our study, we included a total of 629 patient samples and 84,139 image patches, making it that with the highest case number on lymphoma subtyping by deep learning to date.

Currently, the use of a training, validation, and test sets is advocated. The deep learning algorithm is trained and optimized using the first two sets. The test set should only be used for final classification. This setup was used by most investigators on lymphoma, including our own, but not in all previous studies [22].

The patch size of the final images ranged between 16 × 16 px and 800 × 800 px in most studies [31]. Currently, there is no standard regarding the size of the image patches, but it seems fair to argue that, if the size is smaller in terms of cytological features and if the size is larger, the architecture is better represented. Some of the variation in pixel size is due to different magnifications used [18]. Often, images are either extracted at ×200 or, as in our study, at ×400 [22]. In this regard, it is important to note that previous investigations on the classification of follicular lymphoma versus reactive follicular hyperplasia, both processes that show prominent architectural changes, included rather low magnifications to ensure architectural representation [18]. As SLL/CLL and DLBCL show very distinct cell morphologies, we used a higher magnification (400×) to have a better cytological representation of the respective cell types. During the annotation, we tried to avoid a prominent representation of the tissue edge from the tissue cores, thereby ensuring transferability to whole slides. Although not explicitly tested, we would expect our algorithm to achieve similar results on whole-slide images, as the image patches from TMA cores and from whole slides are comparable.

Moreover, different CNN architectures have been applied in previous studies. We decided to use the EfficientNet framework because it achieved a high top-1 accuracy of 84.3% on the ImageNet dataset, while being smaller and significantly faster than network architectures achieving comparably high accuracy rates on the same dataset [34]. The EfficientNet architectures use a compound scaling method to balance width, depth, and resolution of a network, and they have successfully been applied to histopathological image classification tasks [35]. Computational time might be an important factor not only for training models, but also for application in routine diagnostics. In this regard, it would be beneficial to find an equally fast way to use CPUs in a routine context, especially because these have a low inference time and most computers worldwide are not equipped with a GPU.

Lastly, there is also no established standard for evaluation. Most authors used a majority vote where the class with the highest probability was chosen as the final result [22,32]. If multiple magnifications were included, the final result was calculated by averaging the respective single results [18]. We believe that, if multiple classes are included in an algorithm, it might not be enough to calculate the final result on the basis of a majority vote. If, for example, the algorithm is trained on three diseases, the random chance for each class would be 33.3%. In the given example, a doubtful result of 34% probability for one class would trigger this class to be labeled as the final result. The application of quality control limits has previously been proposed and applied [19]. In the abovementioned lymphoma studies, only one group used the quality control limits at the image patch level to create heatmaps at the patient level, but this method was not applied for the final classification result [18]. For applications in the routine diagnostic setting, the implementation of quality control measures is important in our opinion. We tested the effect of quality control limits at the image patch and patient level and achieved not only an increase in accuracy, but also automatic screening for cases with doubtful results that need further review.

In a routine diagnostic scenario, a small and resource-sparing panel of confirmatory immunohistological and/or molecular methods could be ordered after confirmation of the deep learning result by a pathologist. Specifically, in cases where LNs are reviewed for metastasis of carcinomas by pathologists with low expertise in terms of hematological neoplasia, our algorithm could raise alertness for an underlying hematological neoplasm such as SLL/CLL [36].

The limitations of our study are the sample size, the number of included entities, and the process for hyperparameter tuning. Herein, we examined a total of 629 cases. Following the random separation into training, validation, and test sets, only 378 cases were included in the training set. SLL/CLL and DLBCL may both be morphologically different, and many variants and specific morphological features are recognized in the current World Health Organization classification [3]. In this regard, it must be noted that some subsets of SLL/CLL may show extensive plasmacytoid appearance [37], may exhibit large confluent proliferation centers, i.e., not the equivalent of Richter transformation [38], or may show differences in proliferative activity and prognosis according to IGH gene homology with a germline sequence [39]. Likewise, there are specific forms of DLBCL such as the activated B-like and germinal center B-like subtypes that have distinct morphological, immunohistological, and genetic characteristics [40]. As a function of the described variations which are mainly due to distinct molecular changes, it becomes clear that a limited number of cases and extracted image patches per patient can only display a fraction of the overall possible morphological spectrum of SLL/CLL and DLBCL, as well as their reactive changes. Our model was trained to detect only two B-NHLs. Therefore, it cannot be expected that the algorithm will reliably classify other types of B-NHLs, lymphomas of T-cell origin, or Hodgkin lymphomas that were not trained in the current study. Moreover, a small number of tumor cells per image patch may be a limiting factor, and the minimal number of tumor cells per image patch needed for a reliable result is currently not clear. It is possible that the misclassification of SLL/CLL patient samples as normal lymph nodes occurred, even when applying high-quality control thresholds, due to the fact that neoplastic cells represent only a fraction of the overall image area. Our algorithm showed 100% sensitivity and specificity for the detection of DLBCL, but slightly lower sensitivity for SLL/CLL. For screening purposes, it would be desirable to achieve a high sensitivity for lymphoma in order to avoid false negatives, while specificity is less important if additional investigations will be performed. Whereas the introduction of quality thresholds reduced the number of misclassified patients to 3%, the overall problem of lower sensitivity, particularly for SLL/CLL, remained. Considering the abovementioned statements, the application of deep learning for NHL classification must always be conducted under the supervision of a pathologist to avoid misdiagnosis and potentially harmful consequences for patients.

## 5. Conclusions

In the present study, we trained an efficient CNN architecture on scanned histopathological slides and showed that the classification of tumor free LNs, SLL/CLL, and DLBCL is possible with high accuracy. The application of deep learning techniques for histopathological routine diagnostics should be pursued.

## Figures and Tables

**Table 1:** Number of extracted image patches per group.

Group	SLL/CLL	DLBCL	LN Lung	LN Colon	LN Pancreas
Total cases, *n*	129	119	64	230	87
Training set, ~60% of cases					
Cases, *n*	78	80	34	134	52
Image patches, *n*					
Total	11,404	8625	5064	18,488	7004
Minimum	4	7	33	4	2
Maximum	231	278	238	222	245
Mean	146	108	149	138	135
Median	149	99.5	150	142	139
Validation set, ~20% of cases					
Cases, *n*	22	18	15	57	14
Image patches, *n*					
Total	3086	1815	2436	7870	1387
Minimum	3	13	115	24	18
Maximum	214	329	251	255	242
Mean	140	101	162	138	99
Median	146.5	88	156	140	80.5
Test set, ~20% of cases					
Cases, *n*	29	21	15	39	21
Image patches, *n*					
Total	4631	2236	2393	4966	2734
Minimum	18	22	105	17	48
Maximum	226	225	265	184	237
Mean	160	106	160	127	130
Median	189	103	162	132	131

DLBCL: diffuse large B-cell-lymphoma, LN: lymph node, SLL/CLL: small lymphocytic lymphoma/chronic lymphatic leukemia.

**Table 2:** Balanced accuracy (BACC) given different case quality control (CQC) thresholds and patch quality control thresholds (PQC).

CQC Threshold		50%		60%		70%		80%		90%	
PQC Threshold	PQC not met (%)	BACC (%)	CQC not met (%)	BACC (%)	CQC not met (%)	BACC (%)	CQC not met (%)	BACC (%)	CQC not met (%)	BACC (%)	CQC not met (%)
**50%**	0.54	84.48	0.8	88.61	5.6	89.55	8	94.12	17.6	95.56	24.8
**60%**	3.44	83.74	0	87.67	4.8	89.55	8	94.12	16.8	93.75	22.4
**70%**	6.43	85.25	0.8	85.99	3.2	89.64	7.2	94.74	15.2	93.75	21.6
**80%**	10.05	85.32	0	86.79	4	89.64	7.2	92.42	11.2	93.75	20.8
**90%**	15.74	85.25	0.8	85.24	2.4	89.64	6.4	91.3	9.6	93.75	18.4

## Data Availability

Scanned tissue sections can be obtained from the NCT tissue biobank (https://www.nct-heidelberg.de/forschung/nct-core-services/nct-tissue-bank.html, accessed on 1 May 2021). Tissue tiles can be obtained from the corresponding author upon reasonable request. The data cannot be uploaded for open access due to current legislation. The code we used to fit our models can be accessed at https://github.com/AG-Computational-Diagnostic/pacltune-pup, accessed on 1 May 2021.

## Supplementary Materials

The following are available online at https://www.mdpi.com/article/10.3390/cancers13102419/s1: Figure S1: Confusion matrix of the best-performing model in terms of the test data at the patch (left) and case level (right).
