# Supplementary material for: Deep Learning for the Classification of Non-Hodgkin Lymphoma on Histopathological Images
Source: Cancers (Basel). 2021 May 17;13(10):2419. doi: 10.3390/cancers13102419 (PMC8156071; doi:10.3390/cancers13102419)
Supplement: Supplementary file 1 [file cancers-13-02419-s001.zip › cancers-1220808-supplementary.pdf]

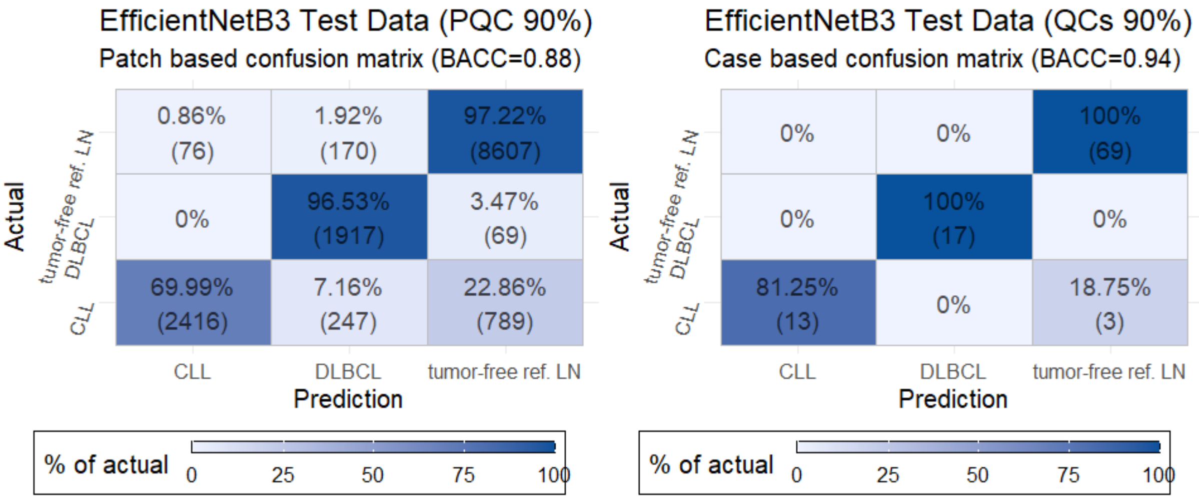

Supplemental Material Figure S1: Confusion matrix of the best performing model in terms of the test data on patch (left) and case level (right). Subtitle exhibits the balanced accuracy (BACC).
